# Supplementary material for: Ultrastable halide perovskite CsPbBr3 photoanodes achieved with electrocatalytic glassy-carbon and boron-doped diamond sheets
Source: Nat Commun. 2024 Mar 30;15:2791. doi: 10.1038/s41467-024-47100-2 (PMC10981704; doi:10.1038/s41467-024-47100-2)
Supplement: Supplementary file 1 — Supplementary Information [file 41467_2024_47100_MOESM1_ESM.docx]

Supplementary Information

**Ultrastable halide perovskite CsPbBr_3_ photoanodes achieved with electrocatalytic glassy-carbon and boron-doped diamond sheets**

Zhonghui Zhu^1,2^, Matyas Daboczi^1^, Minzhi Chen^1^, Yimin Xuan^2*^, Xianglei Liu^2^, Salvador Eslava^1*^

^1^ Department of Chemical Engineering and Centre for Processable Electronics, Imperial College London, London SW7 2AZ, United Kingdom

^2^ School of Energy and Power Engineering, Nanjing University of Aeronautics and Astronautics, Nanjing 210016, China

*Corresponding author, E-mail: Y. Xuan [ymxuan@nuaa.edu.cn](mailto:ymxuan@nuaa.edu.cn), S. Eslava [s.eslava@imperial.ac.uk](mailto:s.eslava@imperial.ac.uk)


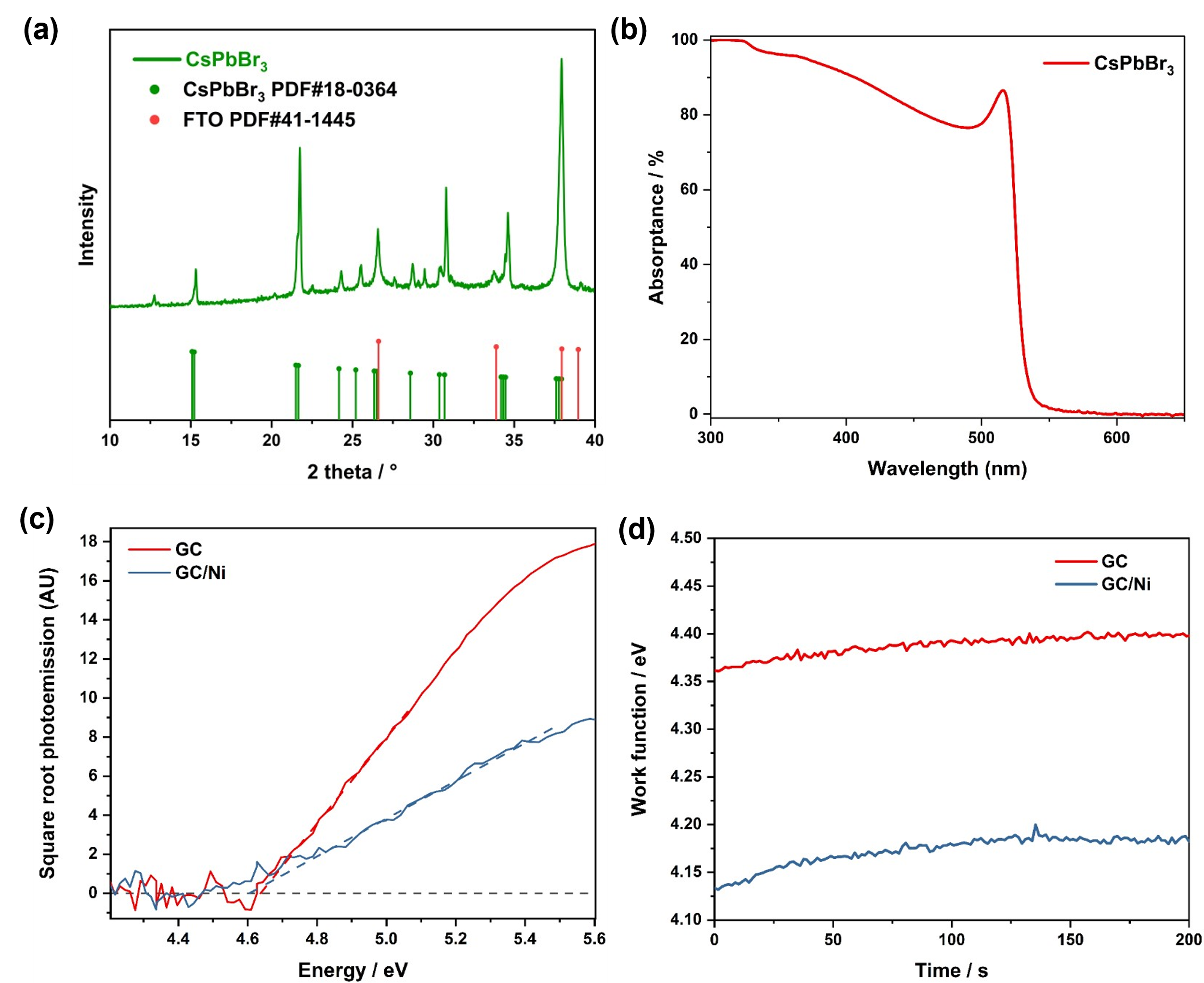


**Supplementary Fig. 1** (a) XRD pattern of the perovskite layer deposited on FTO/SnO_2_. (b) UV-vis absorptance spectrum of CsPbBr_3_ layer on FTO (c) and (d) Square root photoemission spectra and work functions of the bare GC sheet and Ni coated GC sheet.


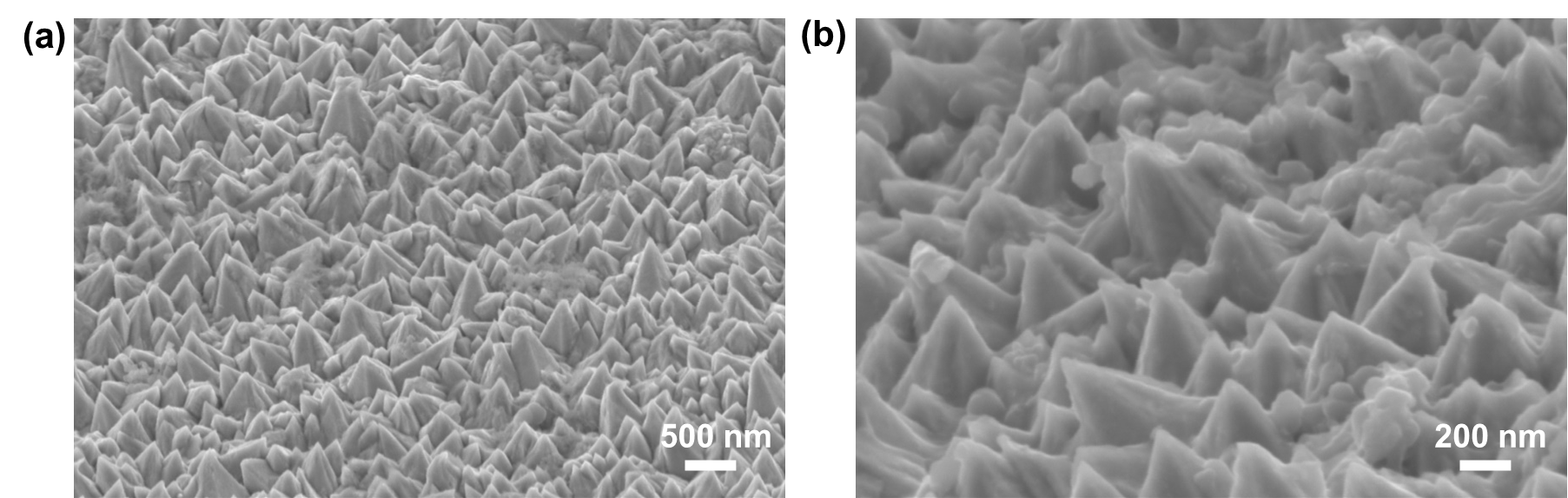


**Supplementary Fig. 2** (a-b) Tilted (45°) SEM micrographs of Ni nanopyramids deposited on a GC sheet. Ni layer is uniformly coated on the surface of the GC sheet. The average base and height of the pyramids is about 300 to 500 nm and 400 nm, respectively.


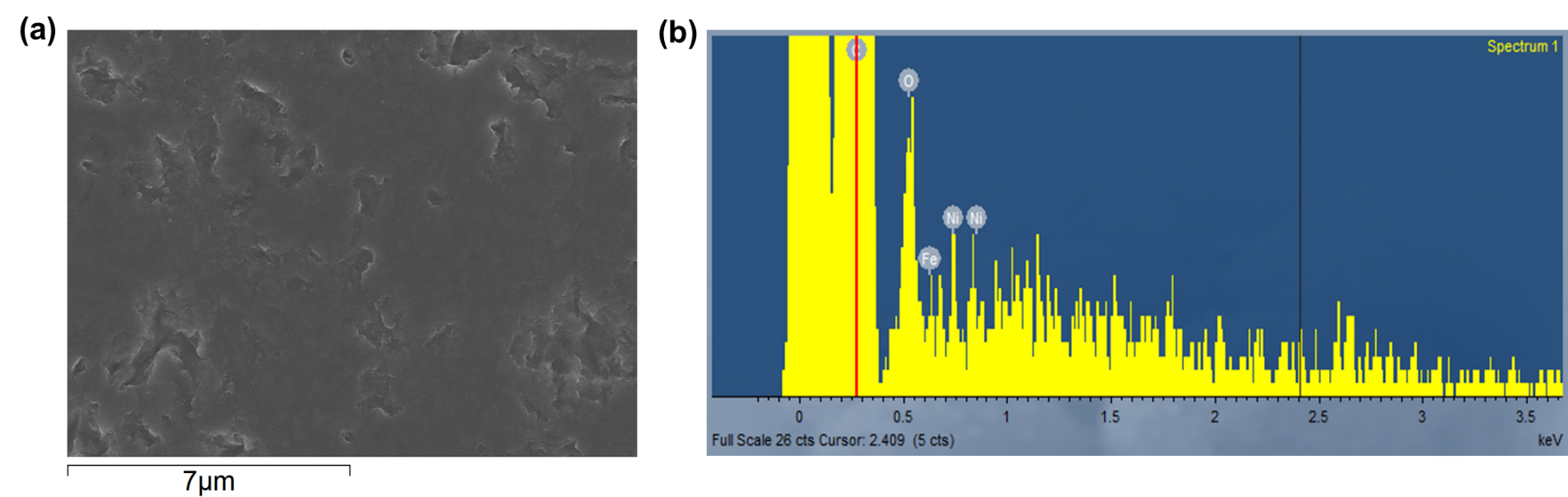


**Supplementary Fig. 3** (a) Top-view SEM micrograph and (b) EDS spectrum of GC/NiFeOOH. The characteristic energy peaks of Ni, Fe, and O elements appear in the EDS spectrum.


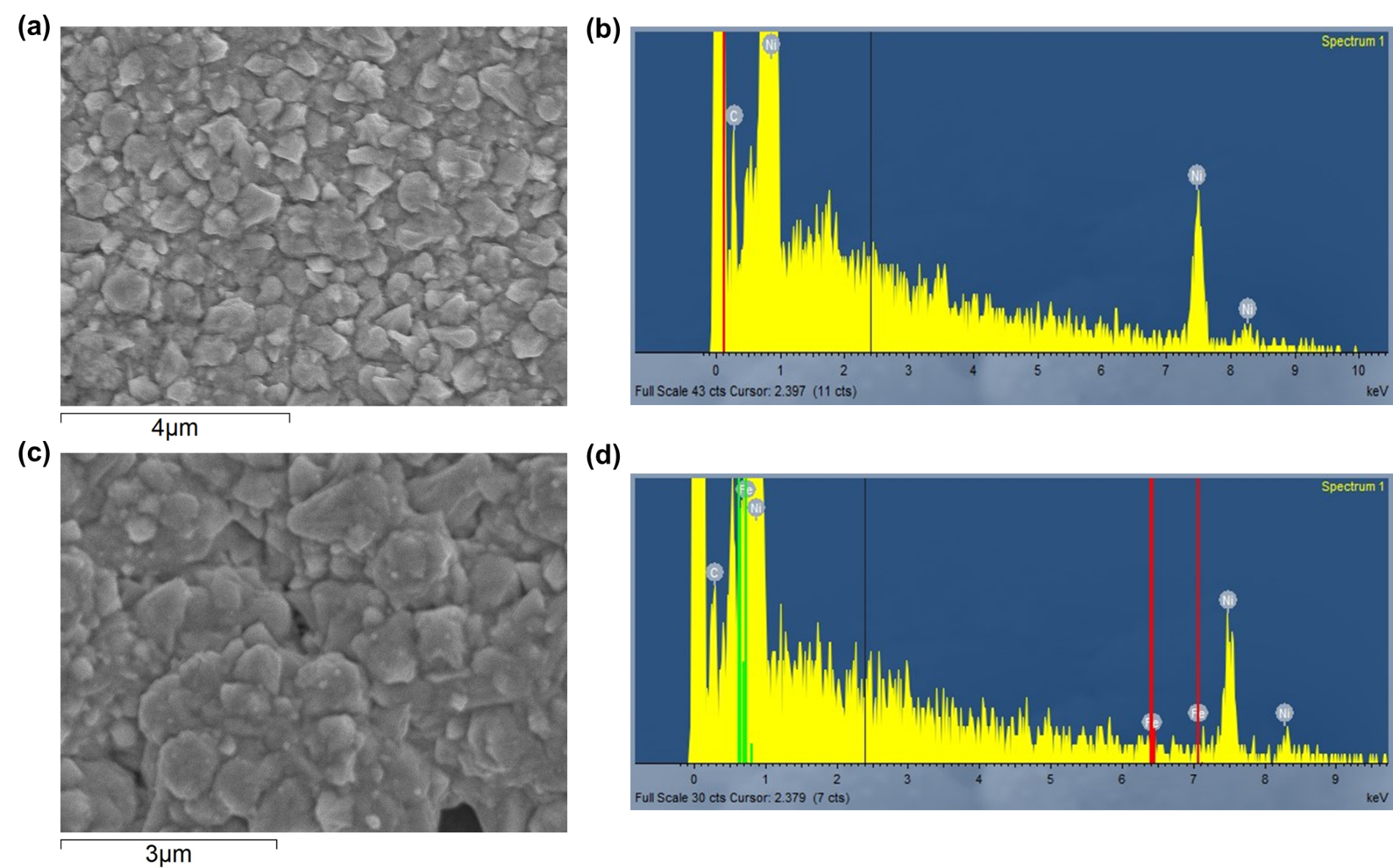


**Supplementary Fig. 4** Top-view SEM micrographs and EDS spectra of GC/Ni nanopyramids (a and b) and GC/Ni-nanopyramids/NiFeOOH (c and d). The EDS spectra show increased energy peak of Ni element in GC/Ni and increased Ni and Fe element peaks GC/Ni-nanopyramid/NiFeOOH. The atomic ratio of C, O, Ni and Fe in GC/Ni is 7.72:6.37:85.91:0 and in GC/Ni/NiFeOOH is 6.55:24.85:65.30:3.30.


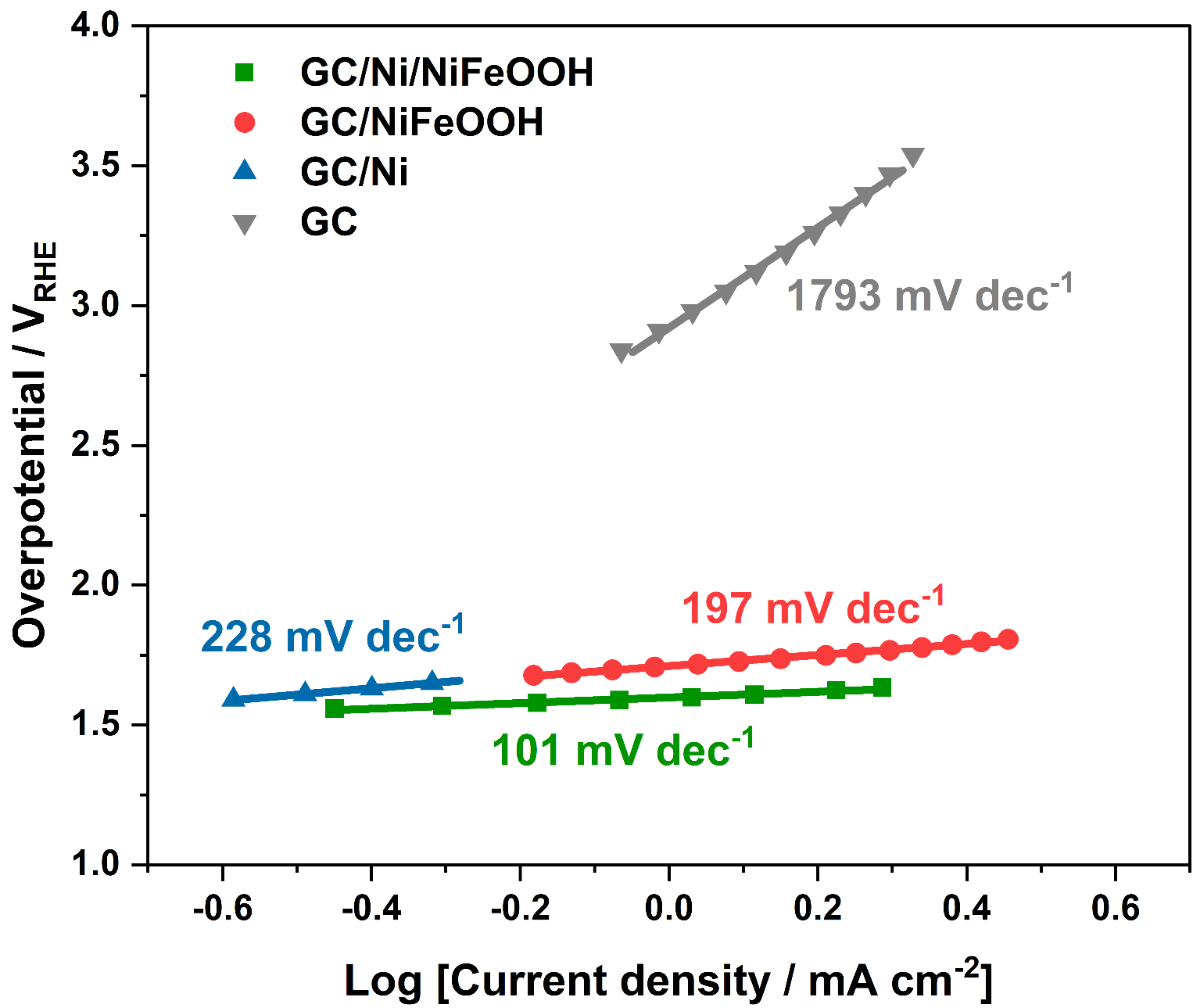


**Supplementary Fig. 5** Tafel plots of bare GC, GC/Ni, GC/NiFeOOH and GC/Ni/NiFeOOH sheets. The Tafel slope of GC/Ni/NiFeOOH (green line) is shown to be remarkably lower than that of bare GC (gray line). \


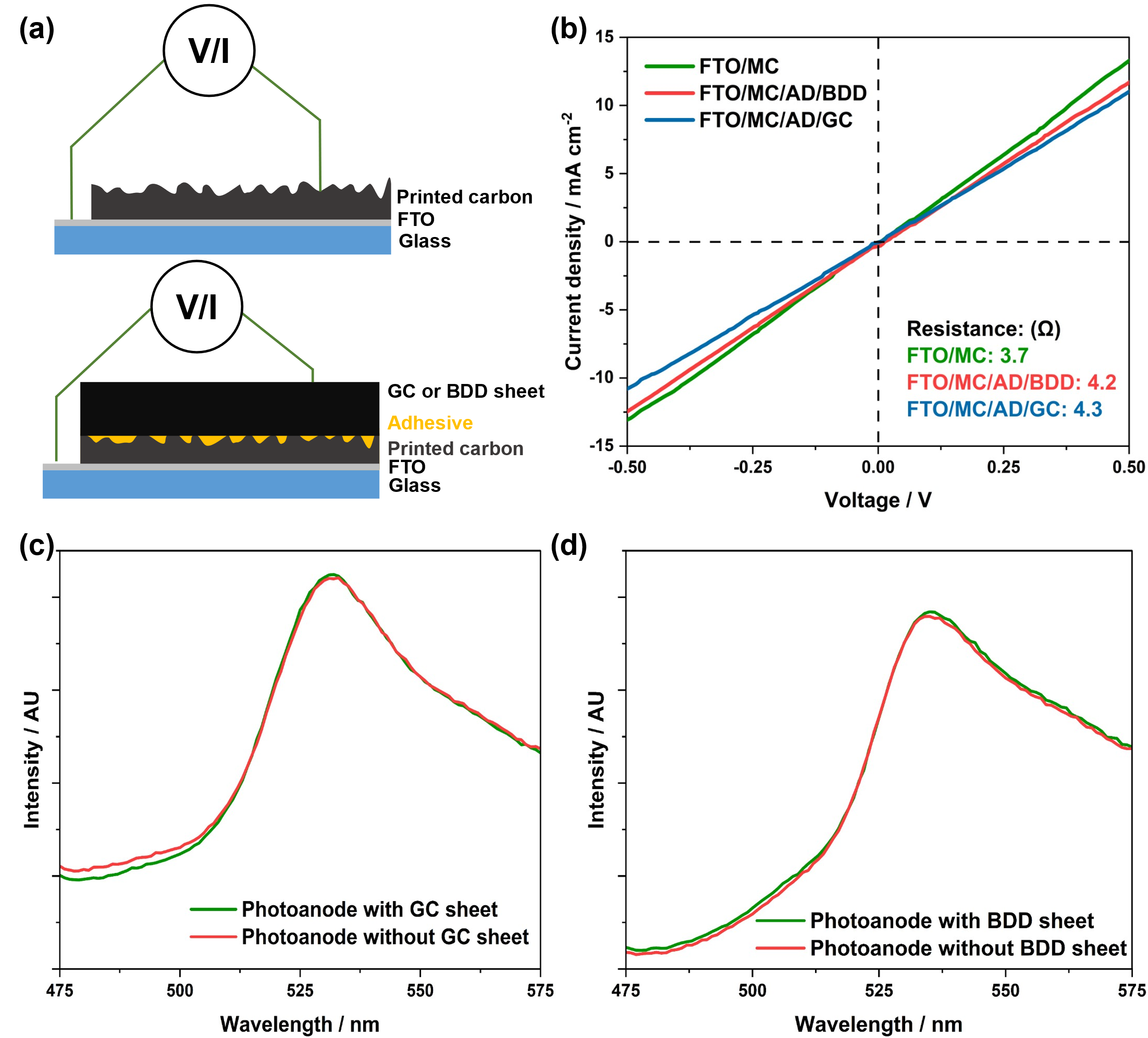


**Supplementary Fig. 6** (a) Sketch of resistance measurement across FTO/mesoporous carbon (MC), FTO/MC/adhesive (AD)/GC, and FTO/MC/AD/BDD interfaces. The printed carbon layer is rough and porous, ensuring the presence of pockets for the adhesive and spikes for the electrical contact to the GC or BDD sheets. (b) J-V scans of these structures to calculate resistance presented in the inset. The area was controlled to be 1 cm^2^ for all the samples. The small 10-20 mV deviation from the origin is assigned to charging/discharging of surface defects on the FTO and its interface with the carbon layer in this measurement. (c-d) Photoluminescence (PL) spectra of the CsPbBr_3_ photoanodes with and without GC and BDD sheets. This PL was measured from the back of the samples with all the transport layers, so the PL intensity is smaller than usual and the shape of PL is less symmetric.


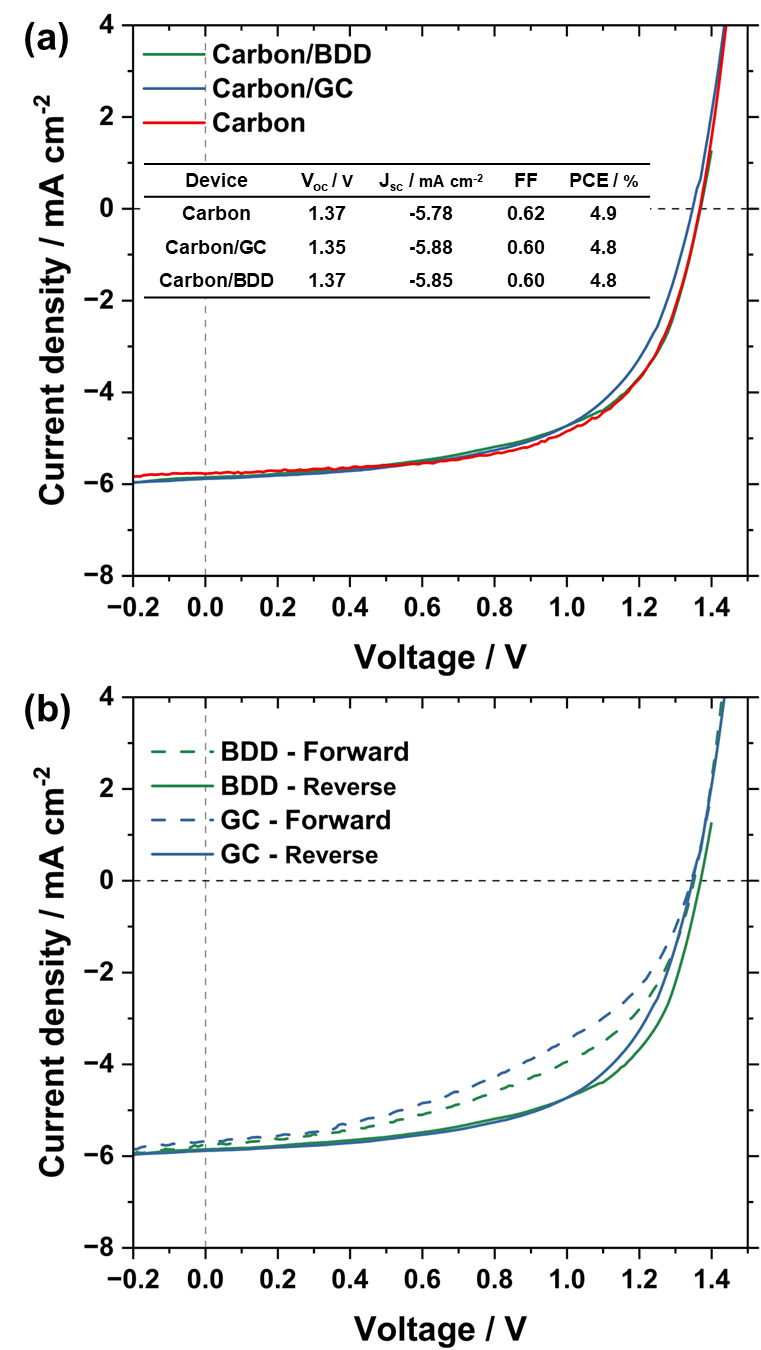


**Supplementary Fig. 7** (a) Reverse current-voltage scans of a CsPbBr_3_ device with and without protective sheets measured as a solar cell. Performance parameters are tabulated in the inset. (b) Forward and reverse current-voltage scans of the same device with either a protective GC and BDD sheet. 1 sun illumination. Scan rate 10 mV s^-1^.


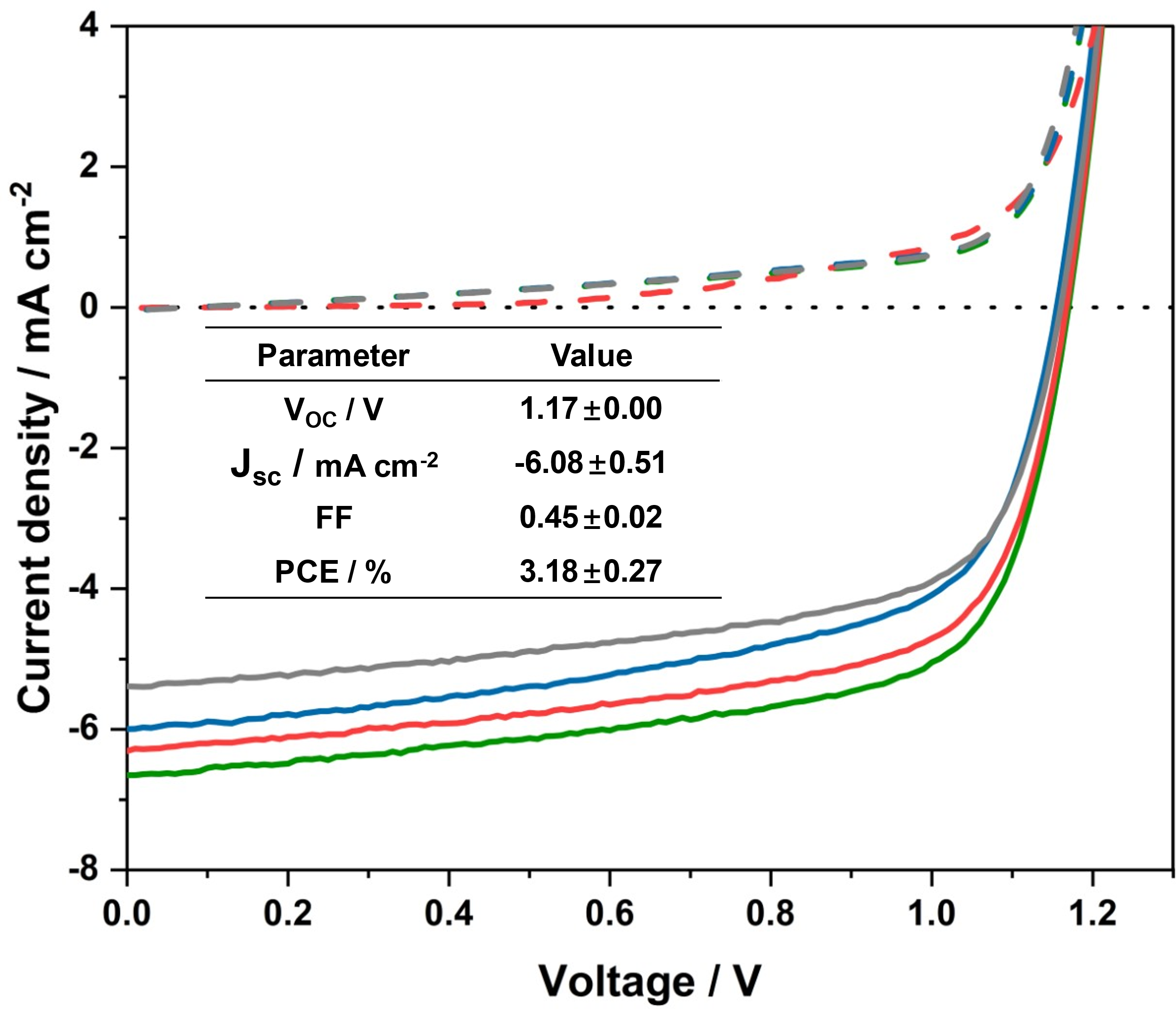


**Supplementary Fig. 8** Reverse current-voltage scans of four CsPbBr_3_ photoanodes with GC/Ni/NiFeOOH sheets measured as solar cells under 1 sun illumination (solid lines) and in dark (dashed lines). Average performance parameters are tabulated in the inset.


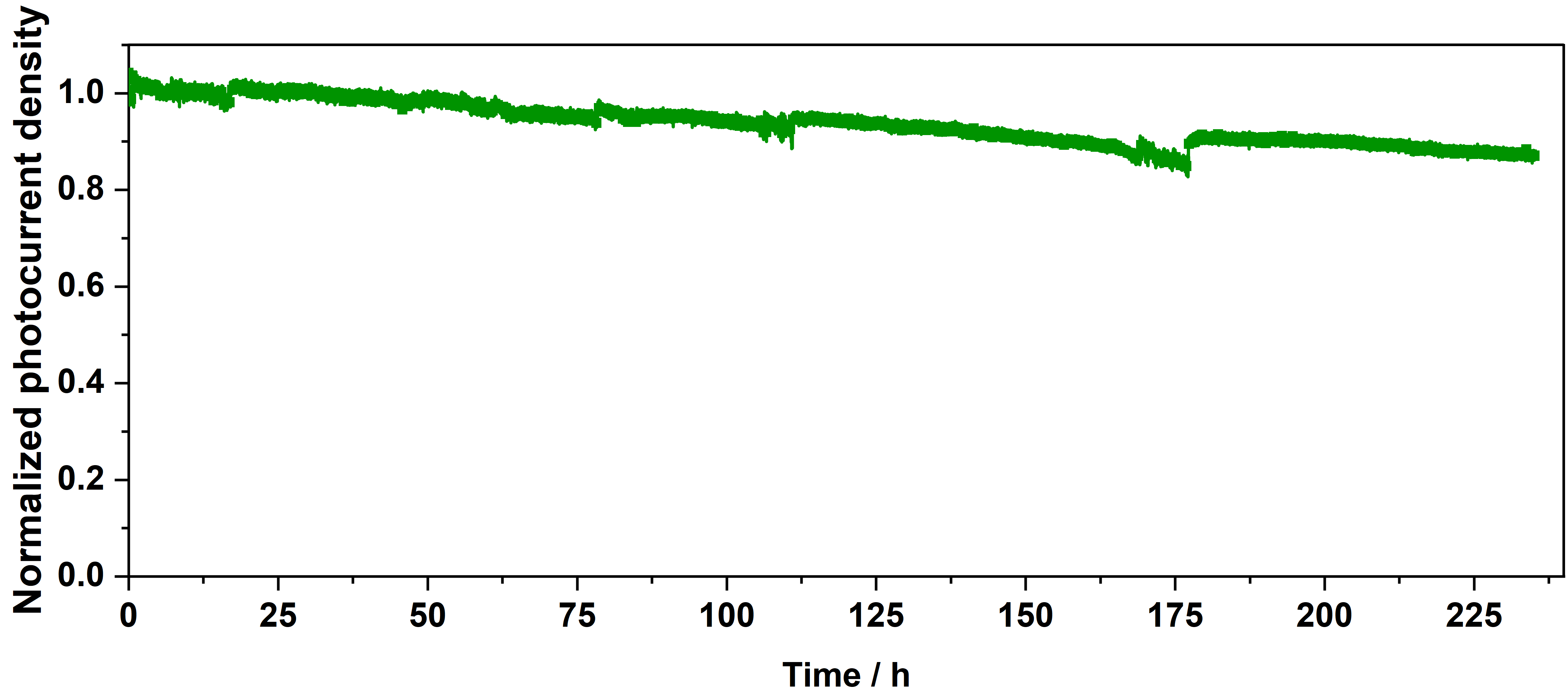


**Supplementary Fig. 9** PV stability at 0.2 V voltage of a CsPbBr_3_ device covered in GC sheet under 1 sun illumination. Initial photocurrent density *j_sc_*: 5.7 mA cm^-2^.


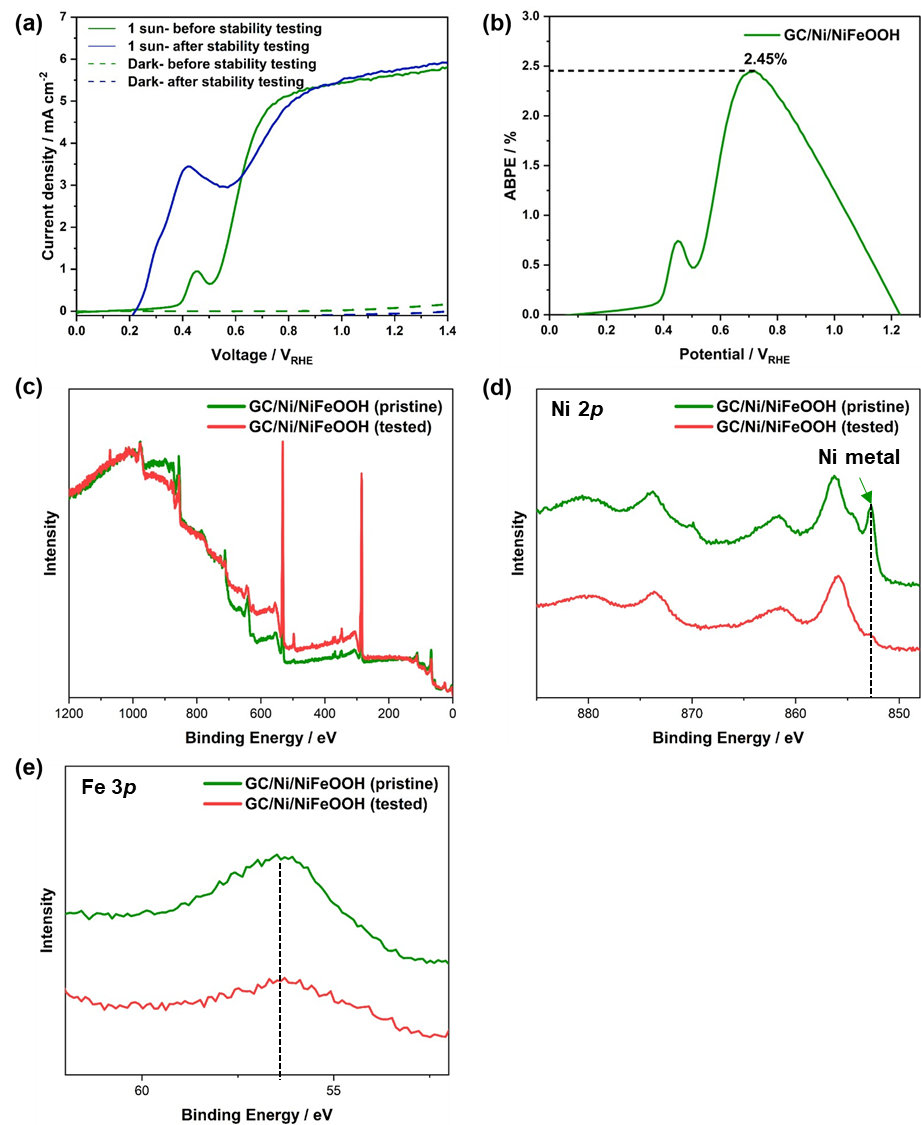


**Supplementary Fig. 10** (a) LSV under 1 sun (solid lines) or in dark (dashed line). (b) Applied bias photon-to-current efficiency (ABPE) of photoanode protected with GC/Ni/NiFeOOH (the data is calculated from the values of Fig. 4a). (c-e) XPS spectra of GC/Ni/NiFeOOH protected photoanode before and after 168 h photoanode stability measurement (presented in Fig. 3d). The peak increase at 0.45 V_RHE_ was caused by the surface activation (i.e., oxidation) of the Ni nanopyramids during the stability test.


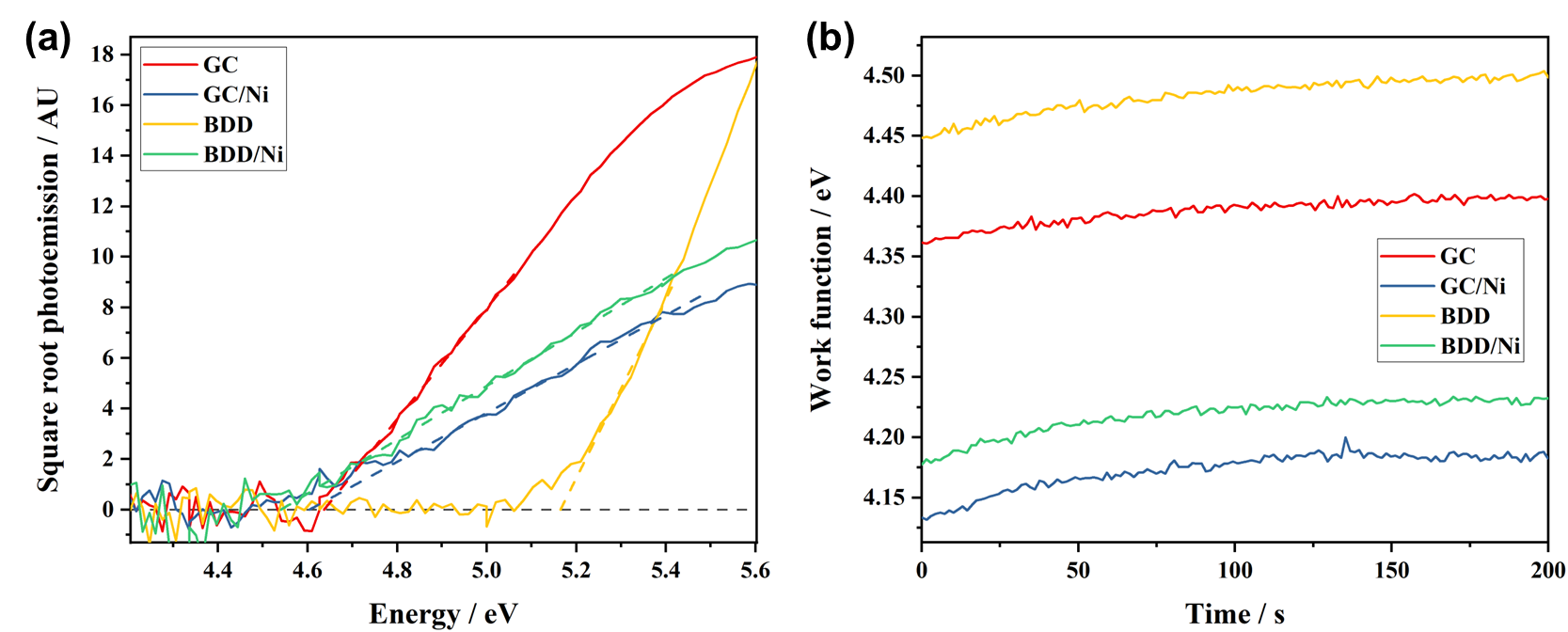


**Supplementary Fig. 11** (a) Square root photoemission spectra and (b) work functions of the bare GC and BDD sheets and Ni coated GC and BDD sheets.

**
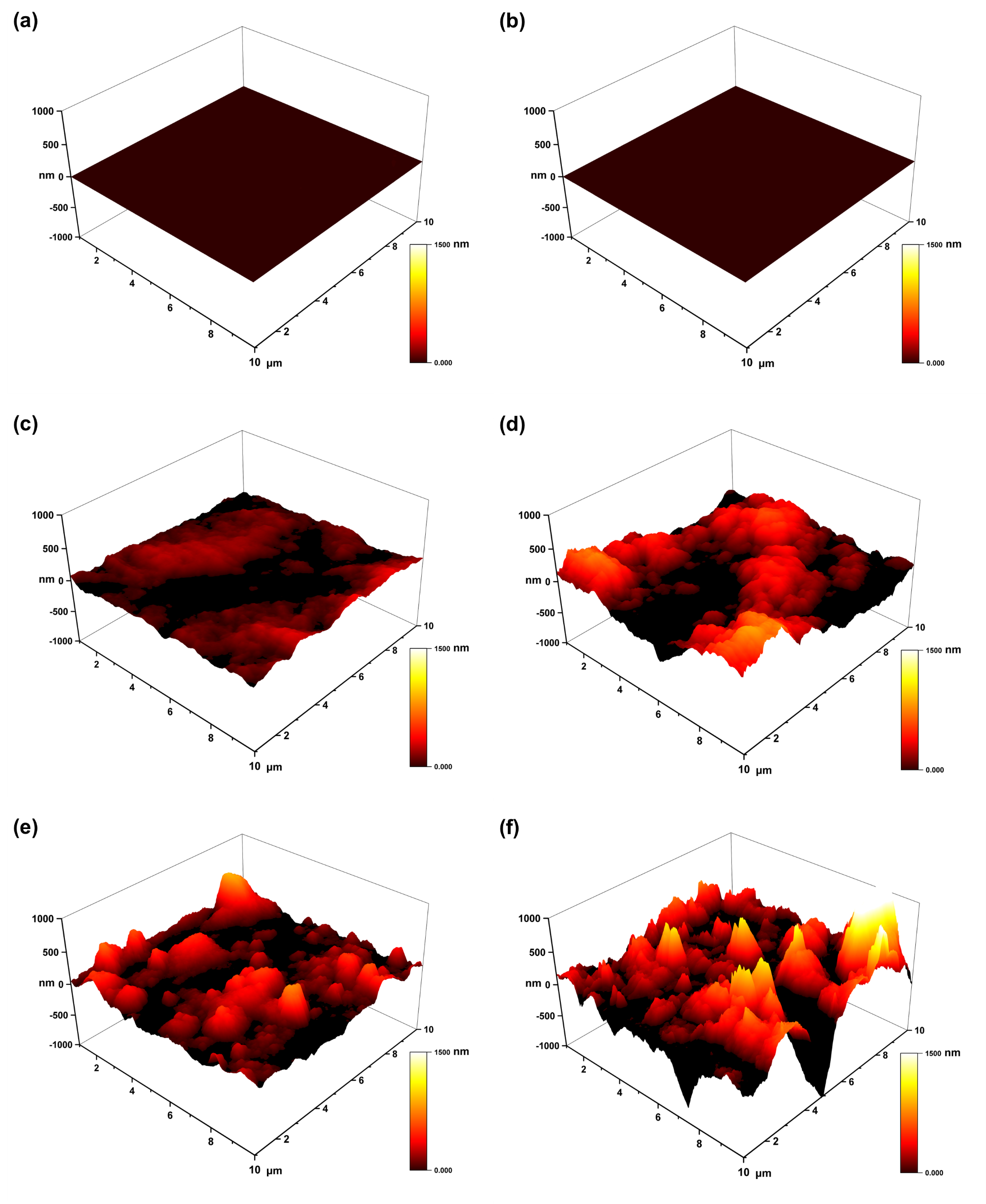
**

**Supplementary Fig. 12** AFM micrographs of (a) back side of GC before adhesive coating, average roughness R_a_ = 0.28 nm, (b) back side of BDD before adhesive coating, R_a_ = 0.31 nm, (c) front side of GC before Ni electrodeposition, R_a_ = 45.7 nm, surface area S_a_ = 1.017 m^2^m^-2^, (d) front side of BDD before Ni electrodeposition, R_a_ = 125.1 nm, S_a_ = 1.084 m^2^m^-2^, (e) GC/Ni/NiFeOOH, R_a_ = 85.5 nm, S_a_ = 1.096 m^2^m^-2^, and (f) BDD/Ni/NiFeOOH, R_a_ = 160.4 nm, S_a_ = 1.561 m^2^m^-2^.


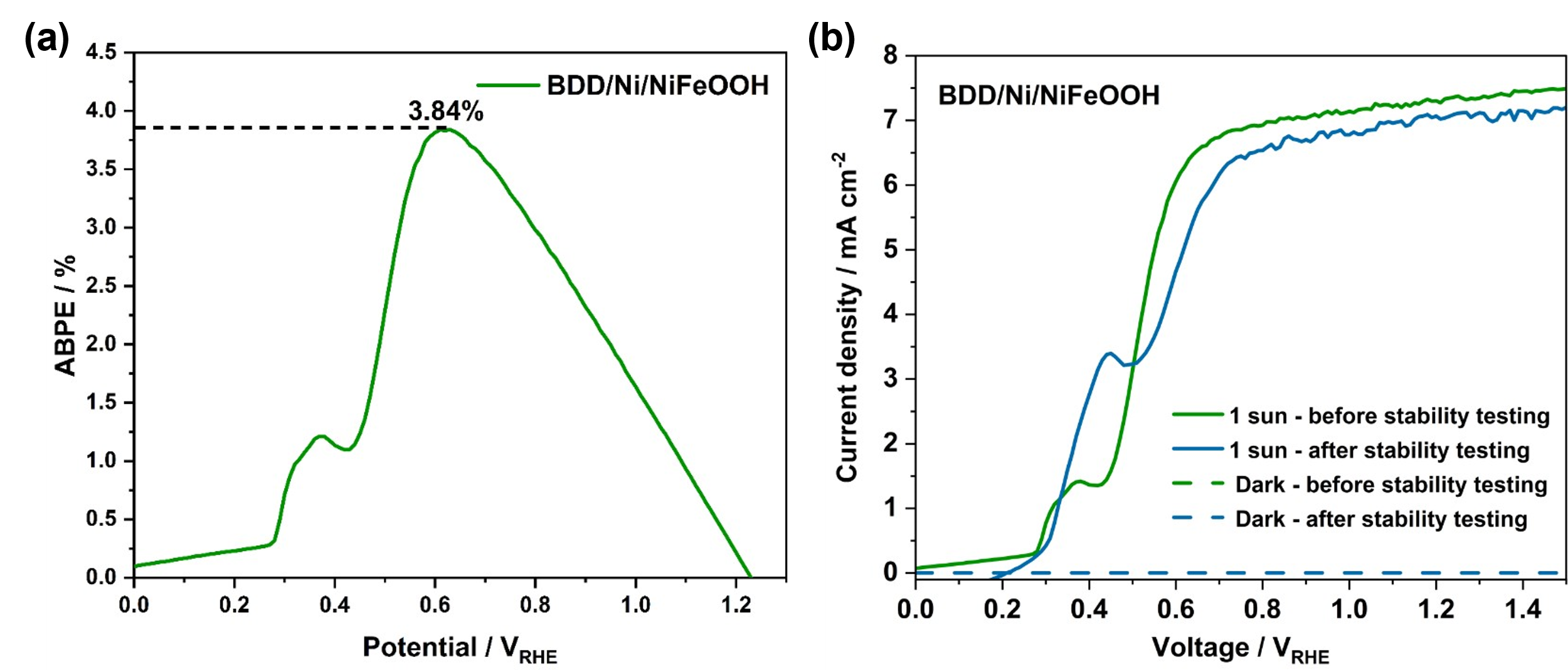


**Supplementary Fig. 13** (a) ABPE of photoanode protected with BDD/Ni/NiFeOOH sheet, calculated from the values of Fig. 5c. The ABPE was calculated by the same equation of Supplementary Fig. 8b. (b) OER polarization scans (50 mV s^-1^ scan rate) under 1 sun illumination and in dark condition of CsPbBr_3_ photoanode with BDD/Ni/NiFeOOH sheet before and after 200 h long-term operation.


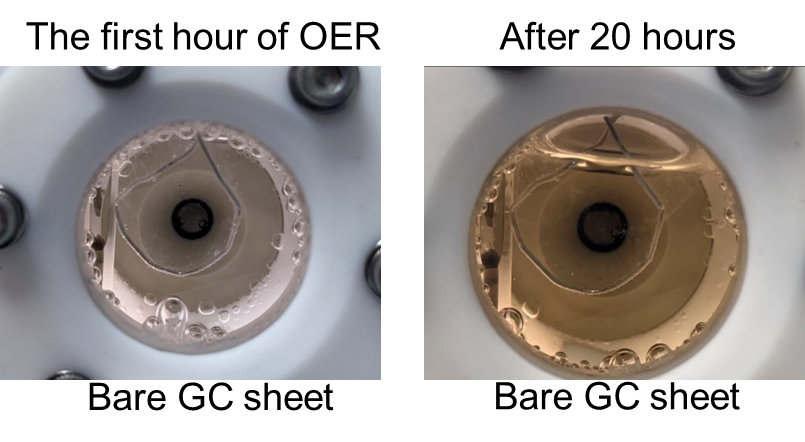


**Supplementary Fig. 14** Photographs of electrolyte in the reaction cell under 1 sun illumination of the CsPbBr_3_ photoanode with bare GC sheet at the first hour and after 20 hours. In the absence of Ni/NiFeOOH, the electrolyte color became yellow due to the degradation of glassy carbon.


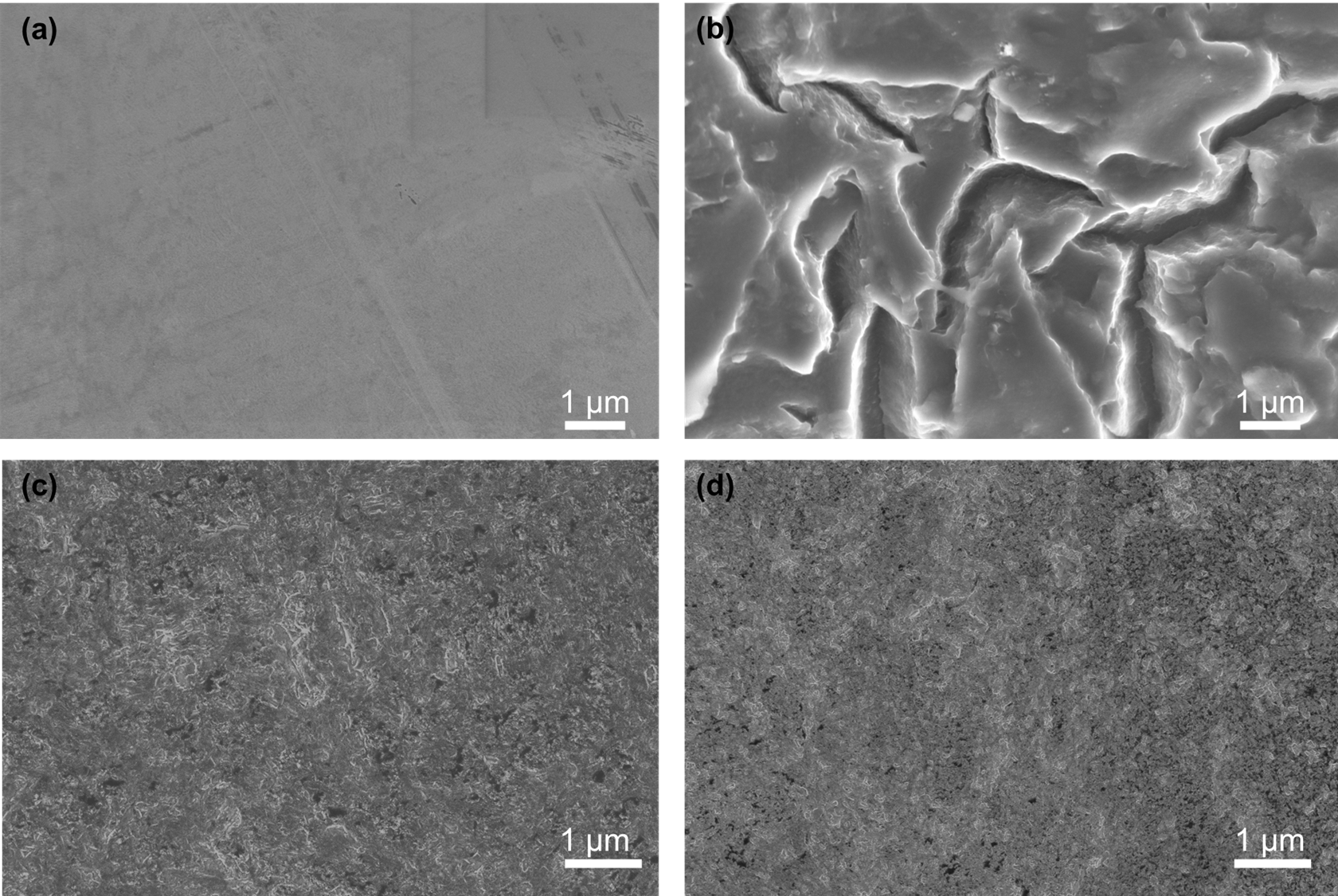


**Supplementary Fig. 15** SEM micrographs of bare GC sheet before (a) and after (b) 64 h long stability measurement. SEM micrographs of bare BDD sheet before (c) and after (d) 70 h stability measurement. The structure of glassy carbon was destroyed and more surface was exposed. The BDD sheet however showed no changes on the surface. Tests carried out in in aqueous 1 M NaOH at +1.23 V_RHE_.


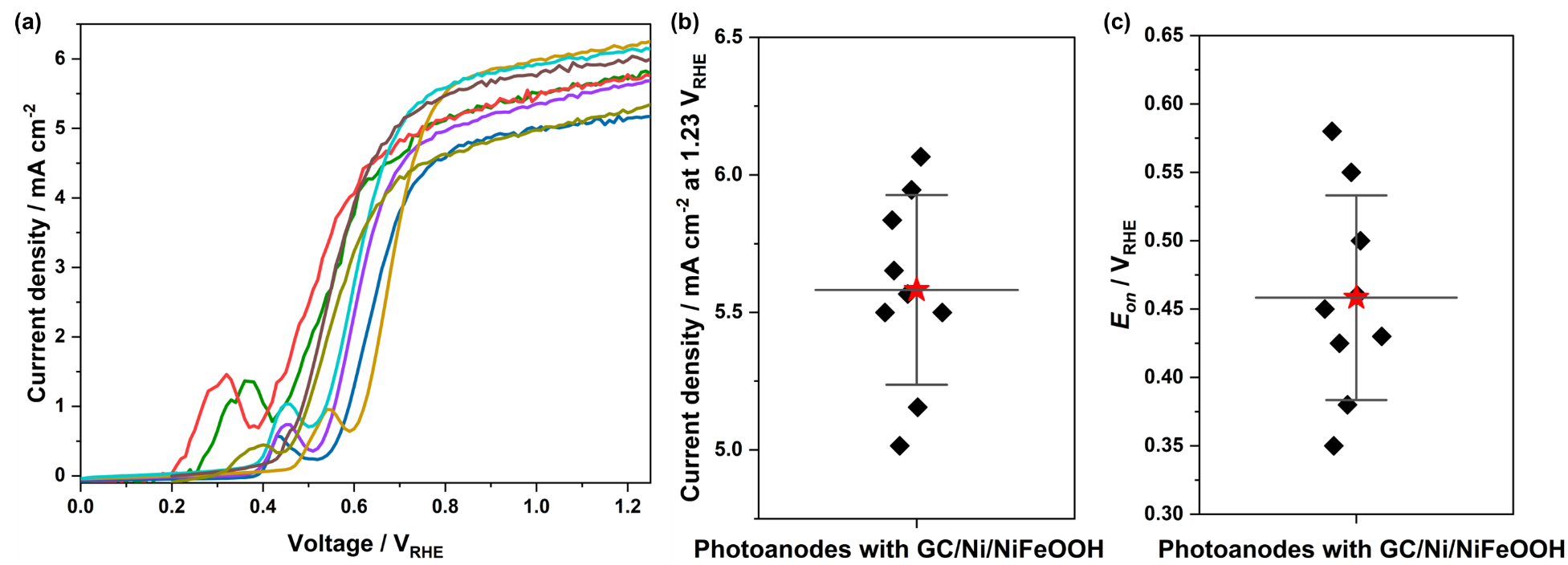


**Supplementary Fig. 16** Statistical analysis of photoanode PEC performance by reusing glassy carbon (GC) sheets. (a) LSV polarization scans for 9 photoanodes by reusing 3 pieces of glassy carbon sheets under 1 sun illumination in 1 M NaOH (all GC sheets were reused 3 times to fabricate photoanodes). (b-c) Box plots of photocurrent densities at +1.23 VRHE and onset potentials (*Eon*) obtained from LSV data in (a). The mean values are represented by red stars, diamond symbols are the experimental data, and the whiskers show one standard deviation.


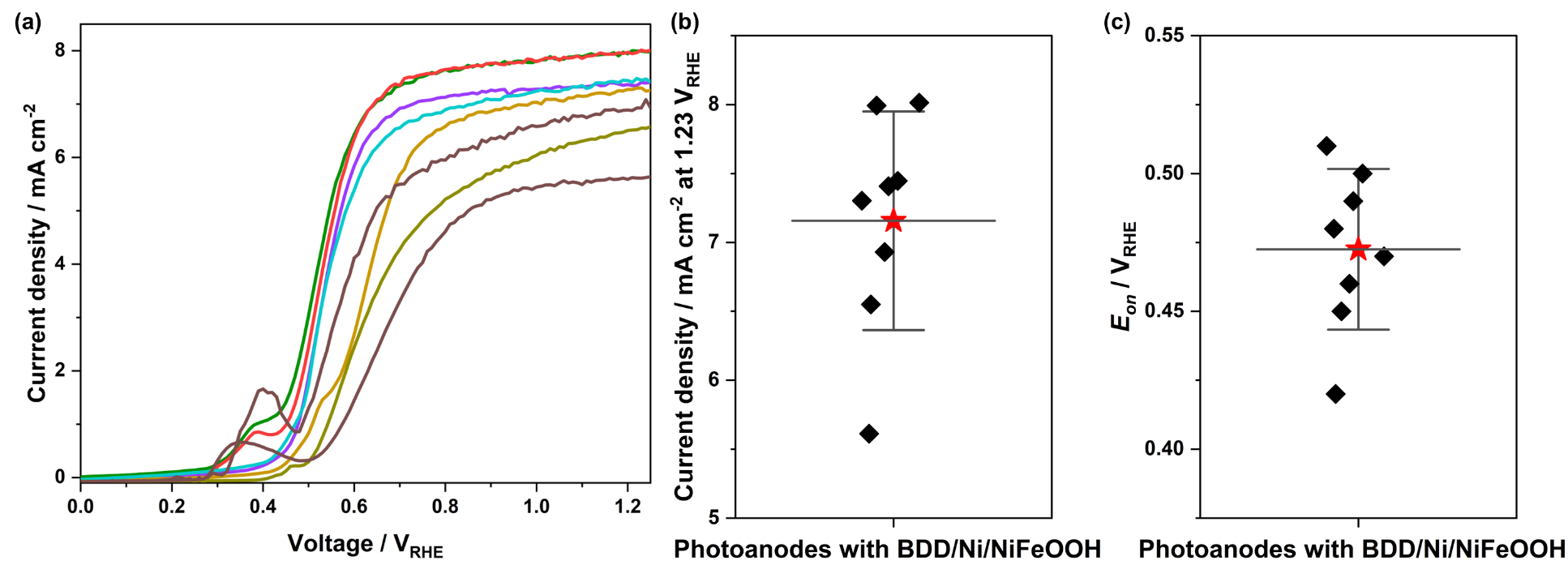


**Supplementary Fig. 17** Statistical analysis of photoanode PEC performance by reusing boron-doped diamond (BDD) sheets. (a) OER polarization scans for 8 photoanodes by reusing 2 pieces of glassy carbon sheets under 1 sun illumination in 1 M NaOH (all BDD sheets were reused 4 times to fabricate photoanodes). (b-c) Box plots of the photocurrent densities at +1.23 VRHE and onset potentials (*Eon*) obtained from LSV data in (a). The mean values are represented by red stars, diamond symbols are the experimental data, and the whiskers show one standard deviation.


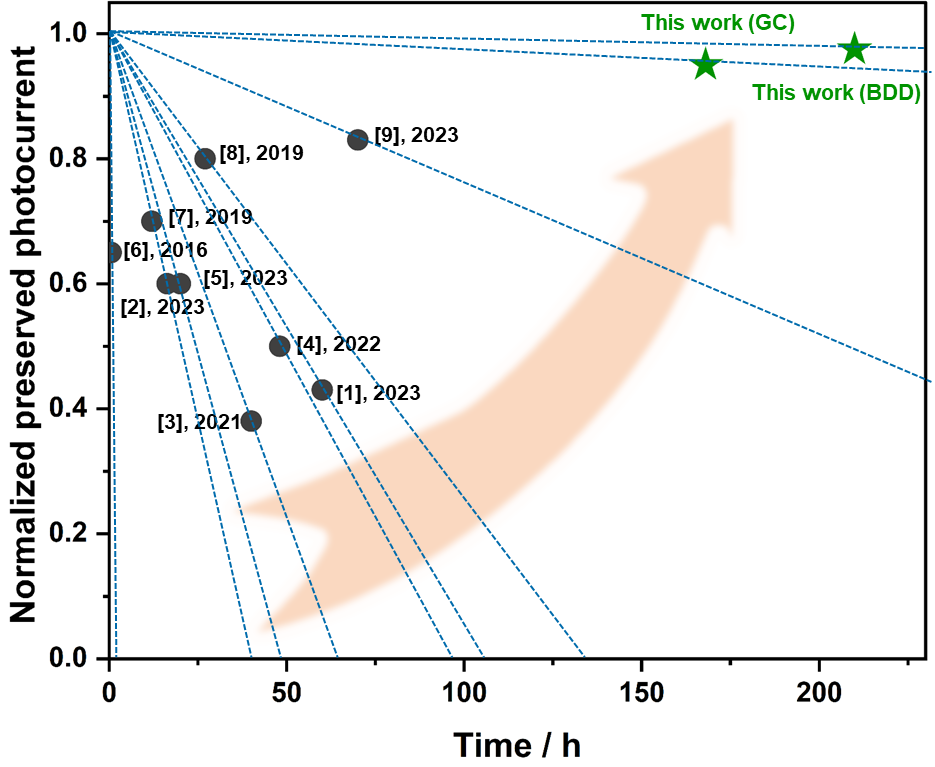


**Supplementary Fig. 18** Comparison of normalized preserved photocurrent among PEC devices including halide perovskite photoanodes for solar-driven OER (AM1.5G, 1 sun). Further details in the Supplementary Table 1. Linear extrapolations in the background are added for visual aid.

**Supplementary Table 1** Comparison of reported photoelectrochemical devices including halide perovskite photoanodes for solar-driven OER (AM1.5G, 1 sun)

| Perovskite  material | Protection layer^1^ | Stability duration test | *J_n_^#^* | Initial max. ABPE | Manuscript Reference |
| --- | --- | --- | --- | --- | --- |
| FAPbBr_3_ | GS/NiFe alloy/NiFe LDH | 60 h | 43% | 8.5% | 1 |
| (FAPbI_3_)_0.97_(MAPbI_3_)_0.03_ | GS/IrOx cat. | 16.3 h | 60%^$^ | 11.3% | 2 |
| FA_0.83_Cs_0.17_Pb(I_0.8_Br_0.2_)_3_ | Field metal/Ni | 40 h | 38 % | 5.8% | 3 |
| FAMAPbI_3_ | Ni/NiFeOOH | 48 h | 50 % | 9.2% | 4 |
| FAMAPbI_3_ | Ni foil/3D Ni.NiFe | 20 h | 60 %^@^ | 8.9% | 5 |
| MAPbI_3_ | Carbon nanotube  /polymer | 0.5 h | 65 % | - | 6 |
| MAPbI_3_ | Carbon | 12 h | 70 % | - | 7 |
| CsPbBr_3_ | GS/Ir mol. cat. | 27 h | 80 % | - | 8 |
| CsPbBr_3_ | dense GS/GS/ /NiFeOOH | 70 h^&^ | 83 % | - | 9 |
| CsPbBr_3_ | GC/Ni/NiFeOOH | 168 h | 95 % | 2.5% | This work |
| CsPbBr_3_ | BDD/Ni/NiFeOOH | 210 h | 97 % | 3.8% | This work |

^#^  *J_n_*: photocurrent density preserved at the end of the stability test compared with its initial stabilized value.

^$^ Measured in a tandem device of photoanode and photocathode, in which the photocathode side is reported to keep about 90 % of the initial photocurrent density upon 60 h operation. The authors also noted that the application of the GS/IrO_x_ and edge encapsulation with epoxy in atmospheric air may accelerate the degradation rate of the Spiro-OMeTAD based hole transport layer in the n-i-p building block (i.e., in the photoanode). The authors also report perovskite/silicon tandem photoanodes protected with GS/IrO_x_ that achieve longer stabilities, but the last layer on the aqueous electrolyte side is silicon, not a halide perovskite.

^@^ Measured in a tandem device of photoanode and photocathode, in which the photocathode side is reported to keep 76 % of the initial current density upon 20 h operation.

^&^ Without graphite sheet replacement.

GS: graphite sheet

LDH: layered double hydroxide

GC: glassy-carbon sheet

BDD: boron doped diamond sheet

## Supplementary References

1 Yang, H., Liu, Y., Ding, Y., Li, F., Wang, L., Cai, B., Zhang, F., Liu, T., Boschloo, G., Johansson, E. M. J. & Sun, L. Monolithic FAPbBr_3_ photoanode for photoelectrochemical water oxidation with low onset-potential and enhanced stability. *Nature Communications* **14**, 5486 (2023).

2 Fehr, A. M. K., Agrawal, A., Mandani, F., Conrad, C. L., Jiang, Q., Park, S. Y., Alley, O., Li, B., Sidhik, S., Metcalf, I., Botello, C., Young, J. L., Even, J., Blancon, J. C., Deutsch, T. G., Zhu, K., Albrecht, S., Toma, F. M., Wong, M. & Mohite, A. D. Integrated halide perovskite photoelectrochemical cells with solar-driven water-splitting efficiency of 20.8%. *Nature Communications* **14**, 3797 (2023).

3 Wang, M., Li, Y., Cui, X., Zhang, Q., Pan, S., Mazumdar, S., Zhao, Y. & Zhang, X. High-Performance and Stable Perovskite-Based Photoanode Encapsulated by Blanket-Cover Method. *ACS Applied Energy Materials* **4**, 7526-7534 (2021).

4 Kim, T. G., Lee, J. H., Hyun, G., Kim, S., Chun, D. H., Lee, S., Bae, G., Oh, H.-S., Jeon, S. & Park, J. H. Monolithic Lead Halide Perovskite Photoelectrochemical Cell with 9.16% Applied Bias Photon-to-Current Efficiency. *ACS Energy Letters* **7**, 320-327 (2022).

5 Rhee, R., Kim, T. G., Jang, G. Y., Bae, G., Lee, J. H., Lee, S., Kim, S., Jeon, S. & Park, J. H. Unassisted overall water splitting with a solar-to-hydrogen efficiency of over 10% by coupled lead halide perovskite photoelectrodes. *Carbon Energy* **5**, e232 (2023).

6 Hoang, M. T., Pham, N. D., Han, J. H., Gardner, J. M. & Oh, I. Integrated Photoelectrolysis of Water Implemented On Organic Metal Halide Perovskite Photoelectrode. *ACS Applied Materials & Interfaces* **8**, 11904-11909 (2016).

7 Tao, R., Sun, Z., Li, F., Fang, W. & Xu, L. Achieving Organic Metal Halide Perovskite into a Conventional Photoelectrode: Outstanding Stability in Aqueous Solution and High-Efficient Photoelectrochemical Water Splitting. *ACS Applied Energy Materials* **2**, 1969-1976 (2019).

8 Poli, I., Hintermair, U., Regue, M., Kumar, S., Sackville, E. V., Baker, J., Watson, T. M., Eslava, S. & Cameron, P. J. Graphite-protected CsPbBr_3_ perovskite photoanodes functionalised with water oxidation catalyst for oxygen evolution in water. *Nature Communications* **10**, 2097 (2019).

9 Daboczi, M., Cui, J., Temerov, F. & Eslava, S. Scalable All-Inorganic Halide Perovskite Photoanodes with >100 h Operational Stability Containing Earth-Abundant Materials. *Advanced Materials* **n/a**, 2304350 (2023).
