# Supplementary material for: Ultrastable halide perovskite CsPbBr3 photoanodes achieved with electrocatalytic glassy-carbon and boron-doped diamond sheets
Source: Nat Commun. 2024 Mar 30;15:2791. doi: 10.1038/s41467-024-47100-2 (PMC10981704; doi:10.1038/s41467-024-47100-2)
Supplement: Supplementary file 3 — Description of Additional Supplementary Files [file 41467_2024_47100_MOESM3_ESM.pdf]

<u>DESCRIPTION OF ADDITIONAL SUPPLEMENTARY FILES</u>

Supplementary Movie 1 : Supplementary video showing the evolution of oxygen bubbles in the photoelectrochemical cell
